# The multifaceted challenges faced by women in the field of inherited metabolic disorders

**DOI:** 10.1186/s13023-025-03604-8

**Published:** 2025-03-05

**Authors:** Livia Lenzini, Sara Bianconi, Giorgia Gugelmo, Vincenza Gragnaniello, Simone Messerotti Benvenuti, Gian Paolo Fadini, Nicola Vitturi

**Affiliations:** 1 Division of Metabolic Diseases, Department of Medicine, Padova University Hospital, University of Padova, Padua, Italy; 2 Hospital Psychology Unit, Padua University Hospital, Padua, Italy; 3 Division of Inherited Metabolic Diseases, Department of Women’s and Children’s Health, Padova University Hospital, University of Padova, Padua, Italy; 4 Department of General Psychology, University of Padova, Padua, Italy; 5 Padova Neuroscience Center (PNC), University of Padova, Padua, Italy

**Keywords:** Inherited metabolic disorders, Gender-based medicine, Care-giver

## Abstract

Inherited metabolic disorders (IMDs) are heritable conditions that affect up to 125:100,000 people worldwide. In addition to severe disabling forms that require continuous and costly assistance in both pediatric and adult patients, some IMDs can have mild forms, with the first clinical signs starting in adolescence or very late in adulthood. In the complex field of IMDs, featuring multifaceted challenges that span from scientific discoveries to patient care, women play a central role in contributing to clinical practice, research, patient advocacy, care, and education. In this narrative review, we focused on the involvement of women in the field of IMDs, highlighting not only their extensive contributions but also the undervaluation of the psychological and emotional tolls paid by women dealing with these diseases. Moreover, from a female-centered perspective, we explored the condition of an adult patient with an IMD to highlight the importance of changing the current approach to the clinical management of these diseases toward a more gender-focused approach.

## The challenges of inherited metabolic disorders

Inherited metabolic disorders (IMDs) are heritable conditions that result in metabolic alterations due to mutations in DNA [1]. In most cases, patients with IMDs have a defective gene that results in enzyme deficiency. In most IMDs, a single enzyme is either not produced by the body at all or is produced in a nonfunctional form [2].

The current number of different IMDs is 1907 (http://www.iembase.org/), and even if they are individually rare, they are collectively common, as up to 125:100,000 subjects have an IMD.

In addition to severe neonatal forms, many IMDs can have mild forms with first clinical signs starting in adolescence or very late in adulthood. The number of adult patients with an identified IMD is predicted to increase because of the improved prognosis of early-diagnosed patients (e.g., neonatal screening), the increased awareness of late-onset manifestations among clinicians and because the number of IMDs is constantly expanding, with new disorders and disease mechanisms being described regularly owing to the efforts of scientific research [1].

However, the diagnosis of IMDs is still complex due to their heterogeneous presentations, which can manifest with clinical signs differing even among individuals with similar genotypes and can lead to misdiagnoses or significant delays in identification [3].

The progression of an IMD can vary widely, ranging from acute, life-threatening crises to chronic severe conditions affecting multiple organ systems. Moreover, even if advancements in medical interventions have increased life and health expectancy in individuals with IMD, for many of them, no effective treatment exists, and they carry severe disabilities that require continuous and costly assistance.

In this complex field, featuring multifaceted challenges, women play a central role, spanning from pioneering scientific discoveries to providing essential patient care and support.

Through this narrative review, we focused on the involvement of women in the field of IMDs, highlighting their extensive contributions to clinical practice, research, patient advocacy, care, and education. Moreover, we explored, from a female-centered perspective, the condition of an adult patient with an IMD.

## Women in IMDs’ research, healthcare and advocacy

Female scientists have made significant contributions to the field of IMDs, driving advancements in the diagnosis, treatment, and understanding of these complex disorders. Contemporary female scientists have continued the legacy left in the past century by pioneer researchers.

One of them was Dr. Rosalind Elsie Franklin (1920–1958), whose work in X-ray crystallography represented the first significant contribution of female scientists to the field of IMDs. In her work, she provided crucial insights into the structure of DNA and allowed deciphering of the genetic code by Watson and Crick, which underpinned genetic research pivotal to IMDs.

Gertrud Hurler (1889–1965) during her training as a paediatrician at Hauner Children’s hospital in Germany published the description of the condition which now bears her name (Hurler syndrome) and it is one of the 11 disorders of the mucopolysaccharidoses (MPH I).

In 1947, Gerty Theresa Cori (1896–1957) was awarded the Nobel Prize in Physiology for her discovery of a metabolic pathway involved in IMDs, subsequently named “the Cori cycle”, in which lactate, produced by anaerobic glycolysis in muscles, is transported to the liver and converted to glucose, which is cyclically metabolized back to lactate in muscles.

Eponymously remembered for her description of Andersen disease (Glycogen storage disease type IV) in 1956, Dorothy Hansine Andersen (1901–1963) was an American pediatrician and pathologist, who was also the first recognizing cystic fibrosis as a disease and creating a test to help diagnose it in 1938.

Another key milestone reached by a female researcher was the discovery of X chromosome inactivation by Dr. Mary F. Lyon (1915–2014). Her work was fundamental to elucidate the genetic basis of many X-linked diseases, explaining genetic variance in women with IMDs, such as Anderson Fabry disease.

In addition to their role in scientific advancement, women are at the forefront of healthcare by acting as genetic counselors, nurses, and physicians and providing critical care for patients with IMDs. They often take a holistic approach to patient management, addressing both medical and psychosocial aspects. Genetic counselors, many of whom are women, play a crucial role in guiding families through the complexities of IMDs, offering support and information regarding genetic testing, disease management, and family planning [4].

Women have been instrumental in advocating for better resources, awareness, and policies for IMDs. They lead numerous patient advocacy organizations that work toward improving the lives of those affected by these diseases and actively organize campaigns for information, patient rights, access to treatments and research funding.

Searching for the number of women currently involved in the main European and Italian organizations dealing with rare diseases and, more specifically, with IMDs, we found a robust female presence in roles ranging from executive leadership to grassroots advocacy. For example, the staff of EURORDIS– Rare Diseases Europe (https://www.eurordis.org), the nonprofit alliance of over 1000 rare disease patient organizations from 74 countries, is represented mainly by women (40 of 49 members). Among the 38 patient organizations in the MetabERN, 27 are headered by a woman (https://metab.ern-net.eu/patient-organisations/). Similarly, in Italy, women play a pivotal role in organizations such as UNIAMO, the Italian Federation of Rare Diseases, and most of the IMD patients’ organizations.

Moreover, through their efforts in education and public outreach, women involved in patient associations have significantly increased the awareness of IMDs. Educational initiatives led by women aim to inform both the public and healthcare professionals about the early diagnosis and management of these diseases. Such efforts are crucial for reducing diagnostic delays and improving patient outcomes.

## Women as family caregivers in IMDs

A family caregiver (FC) is any relative or partner who provides a broad range of assistance for a person with a chronic or disabling condition. They are also called informal carers since they assist a person with an unpaid role in general attendance at his/her activities, thus spending a relevant number of hours providing care [5]. Given the wide range of severity of IMDs, many of these disorders strongly impact the life of patients and require constant assistance from FCs not only in children but also in adults.

This support may include demanding daily routines for personal care (e.g., dressing, bathing, eating, etc.), nursing or household activities (e.g., cleaning, cooking, etc.) or mobility and logistic support activities (e.g., help with contact with doctors, financial affairs, insurance, etc.). Moreover, FCs support IMD patients by sharing the psychological and emotional impact of the complexity of clinical management, the often long-lasting wait to receive a diagnosis and the uncertainty of the future if a cure is not yet available. In fact, in most cases, IMDs are chronic and debilitating conditions with no effective therapy, requiring chronic and costly assistance. Moreover, as for all rare diseases, a consistent percentage of patients with IMDs waits until five years to receive a final diagnosis [6].

Given the psychological and emotional impact of caring for an IMD patient, the implications of genetic aspects should also be considered, as the FC may be a carrier of the disease. This could lead to feelings of guilt for the transmission of the IMD [5]. In addition, in the pediatric context, FCs could report stress for the social lives of their children, particularly related to exclusion in social settings [7]; they may also have concerns about their other children, and, somewhat unusually but not uncommonly in genetic disorders, more than one child in the same family could be affected by IMDs [8]. In the last two decades, a relationship has been shown between FCs’ mental and physical health and caregiving activities because of high levels of chronic stress [9]. Accordingly, FCs may develop depression, anxiety, worse sleep quality, and poor physical health more frequently than the general population does [10]. The caregiver burden has been defined as “the extent to which caregivers perceive that caregiving has had an adverse effect on their emotional, social, financial, physical, and spiritual functioning” [11].

Being female has been considered a risk factor for becoming a caregiver and for caregiver burden [12]. In fact, as in the general population (https://www.iss.it/fisiologia-patologia/-/asset_publisher/7z3e64a3XwgM/content/differenze-di-genere-e-salute-nei-caregiver-familiari), where the majority of FCs are 45–55-year-old women, the FCs in IMDs are predominantly women. For example, in a study considering pediatric patients, 18/21 FCs were mothers [7], and in the setting of both pediatric and adult patients with Pompe disease, which is a rare progressive neuromuscular disorder, the majority of FCs were female (60%) [13].

Moreover, even if only a few studies reporting the gender imbalance of the FCs involved in IMDs are available, a generalization can be performed considering all rare diseases. In 2023, Chiarotti F et al. assessed the sex distribution of FCs in patients with ten different rare diseases and reported that 72% of them were female [14]. Another study focused on the perceived burden of informal care for patients with rare diseases in six European countries, and the majority of reported FCs were women (80.2%) [14].

However, the gender imbalance should be considered not only in terms of the number of female subjects playing the role of the prevalent FC but also in terms of the different impacts of patients caring on the lives of female and male FCs. In this context, women, who are more frequently retired early from work, not occupied or homemakers than men, suffer a greater burden than men under the same conditions do [4]. In particular, the high number of hours of every day care for patients with a rare disease may lead to caregiver burden, with consequences for individual and social functioning, which include putting one’s career aside, social isolation, depressive symptoms and poor general health care [15]. Moreover, among pediatric IMD patients with devasting diseases such as mitochondrial disorders, mothers of children with mitochondrial disease had significantly greater caregiver burdens and poorer health-related quality of life, particularly related to role limitations, vitality, and mental health [16].

Thus, women caring for IMD patients encounter many challenges that significantly impact their lives and, often, do not allow them to balance caregiving duties with other responsibilities, such as employment, leading to high levels of stress and burnout. Moreover, they may face financial instability, as many female caregivers reduce work hours or leave their jobs entirely to provide necessary care. Additionally, the lack of sufficient support systems and inadequate recognition of their role cause emotional and psychological strain due to the demanding nature of caregiving, leaving them feeling isolated and overwhelmed and further exacerbating their challenges.

## Women with an IMD

With recent improvements in the diagnosis and clinical management of patients with IMDs, an increasing number of women with these disorders are reaching reproductive age [17]. Thus, pregnancy in these patients can be considered a measure of positive outcomes and transition into adulthood. However, given the biological implications of pregnancy, delivery and the puerperium period, particular attention should be given to pregnancy planning [18]. In particular, in this group of women, pregnancy and the perinatal period involve different issues related to the IMDs [19, 20], such as disease effects on fertility, teratogenic effects of the disease (such as phenylketonuria) [21] or of the therapies, modifications of nutritional treatments [17], worsening of metabolic control [22] or of the underlying maternal IMD due to pregnancy, special recommendations for breastfeeding and IMD effects on labor [23, 24].

Postpartum decompensation may occur in certain metabolic conditions, particularly urea cycle disorders, with behavioral changes due to hyperammonemia potentially resembling symptoms of postpartum psychiatric conditions such as psychosis or depression, highlighting the need for careful monitoring [25].

As another example, clinical manifestations of Acute Hepatic Porphyria (AHP) are also strictly dependent to reproductive hormones and women with acute porphyria seem most susceptible to an acute attack during early pregnancy and the puerperium [26]. However, due to improvement in diagnosis and patient’s awareness, the incidence of complications of pregnancy in AHP is decreasing [27].

Therefore, women with IMDs can have concerns about planning for pregnancy [8], but few studies exist concerning the psychological issues related to pregnancy and puerperium in women with IMDs, and further research is needed.

However, female health extends far beyond reproductive health, as it encompasses specific issues related to physical, mental, and emotional status [28]. In fact, many important aspects of a disease differ between men and women in terms of prevention, clinical manifestations, diagnostic and therapeutic approaches, prognoses, psychosocial effects, and interactions with the health-care system [29]. The application of an approach based on the patient’s sex, as suggested by the World Health Organization (https://www.who.int/health-topics/gender#tab=tab_1), to patients with IMDs would likely overcome many of the challenges related to IMDs in women.

Anderson Fabry disease (AFD) represents a paradigm of the progress achieved by the application of sex medicine [30] to an IMD. This disease is a rare lysosomal storage disorder caused by mutations in the *GLA* gene on the X chromosome, leading to a deficiency in α-galactosidase A (AGAL) enzyme activity and the accumulation of globotriaosylceramide (Gb3) in tissues.

While AFD affects both men and women, the impact on women has often been underrecognized owing to the historical perception that they are only carriers of the disease. However, recent studies have shown that AFD women with one mutated copy of *GLA* also present a wide range of symptoms, from mild to severe, including pain, gastrointestinal issues, kidney dysfunction, heart problems, and stroke [31–34]. This clinical heterogeneity is related directly to the variability in the rate of Gb3 accumulation and impacts the onset of symptoms. In fact, the different degrees of symptom severity in AFD women are due to the random inactivation of the X chromosome, by which, in each body cell, one of the two X chromosomes is randomly inactivated. This phenomenon, discovered by Mary F. Lyon, explains the significant phenotypic heterogeneity among women and the evidence that the activity of AGAL in affected women can range from low to normal [35]. Thus, as lyonization complicates diagnosis, the gold standard for a diagnosis of AFD in female patients is the search for mutations in the *GLA* gene, but a quest for a more affordable biomarker emerged in the diagnosis of the disease in women [36]. Collectively, these issues, together with the lack of awareness among clinicians, lead to a late diagnosis of AFD in women, with up to 15-year diagnosis delays and a subsequent delay in starting the available treatment [31].

Other concerns related to females with AFD, as well as with other X-linked conditions, involve their reproductive age, and they entail the psychological status of AFD women, who feel guilt for transmitting the disorder to their offspring [37]. Furthermore, pregnancy appears to be at risk of complications due to potential microvascular damage to endothelial cells and Gb3 accumulation in both maternal and fetal placental tissues [12, 13].

Thus, even if the management of women with AFD still has some gaps, the progress achieved thus far by applying a gender-focused approach highlights the benefit that a generalization of this model to other IMDs would provide.

For example, in other X-linked disorders, such as ornithine transcarbamylase (OTC) deficiency, the wide range of phenotypic variability in females with a mutant disease-causing allele [39] resembles all the issues described in AFD and, likely, the management of female subjects would improve considering the lesson from AFD.

Due to the X-linked inheritance, also women with an *ABCD1* mutation causing X-linked Adrenoleukodystrophy (ALD) have long been assumed to be merely carriers and to remain asymptomatic. However, in the last years, it has been observed not only that 20–50% of them may develop some symptoms during their lifetime [40–42], but also that X-ALD manifests differently in men and women. In fact, women have no risk of developing the cerebral form of X-ALD but face an 88% risk of developing signs of adrenomyeloneuropathy by the age of 60 [3].

Finally, an intriguing example of the complexity associated with X-linked IMDs is Mucopolysaccharidosis Type II (MPS). In contrast to females with AFD, most female carriers of MPS II do not exhibit symptoms or high urinary levels of glycosaminoglycans [43]. This observation has led to the hypothesis that, in MPS II females, the functional enzyme is secreted by cells expressing the non-mutated gene and is internalized by cells with a non-functioning enzyme, thereby leading to cross-correction of the enzymatic deficiency [44], which has not been observed in AFD.

## Conclusion

The role of women in inherited metabolic diseases is multifaceted and vital. Their contributions as health care professionals, researchers, advocates, and educators have profoundly impacted the understanding, management, and awareness of these diseases. However, the psychological and emotional tolls on women in caregiving are still underrecognized and psychological issues related to reproductive health have not been entirely explored yet. Thus, policies and programs supporting female caregivers, recognition of their work and further research on their psychological needs in pregnancy planning are essential for advancing the field and improving patient care providing appropriate support.

Finally, as shown by the peculiarity of AFD in women, the importance of changing the current approach to clinical management toward a more gender-focused approach is becoming an urgent need in the IMD field.

## Data Availability

All data supporting the findings of this study are available within the paper.
